# Supplementary material for: Rapid turnover of life-cycle-related genes in the brown algae
Source: Genome Biol. 2019 Feb 14;20:35. doi: 10.1186/s13059-019-1630-6 (PMC6374913; doi:10.1186/s13059-019-1630-6)
Supplement: Supplementary file 3 — Protein alignments, corresponding Gblocks html file and CDS sequences for the lineage conserved gametophyte-biased genes. (ZIP 2109 kb) [file 13059_2019_1630_MOESM3_ESM.zip › Con_GA_alignments/Ec-15_004080.aa_ali.fasta-gb.htm]

Ec-15\_004080.aa.fasta.trans.aa\_ali.fasta


## Gblocks 0.91b Results

Processed file: **Ec-15\_004080.aa.fasta.trans.aa\_ali.fasta**  
Number of sequences: **2**  
Alignment assumed to be: **Protein**  
New number of positions: **356** (selected positions are underlined in blue)

```
                         10        20        30        40        50        60
                 =========+=========+=========+=========+=========+=========+
Ec-15_004080     ------------------------------------------------------------
MSTRG.19393.1    GARVLVAQLASDDESLRRTAARVVRGLSRSPYTTSKSLMDADIVRFLLCLLEEGDEPADE
                                                                             


                         70        80        90       100       110       120
                 =========+=========+=========+=========+=========+=========+
Ec-15_004080     ------------------------------------------------MLKALERMAGSP
MSTRG.19393.1    QEEMLCIAYFTKVSSDEEACSELAELGLSRYLIRVLPERRLSLQNRLAMLEALERMAGSP
                                                                 ############


                        130       140       150       160       170       180
                 =========+=========+=========+=========+=========+=========+
Ec-15_004080     RCEKRLIDEGLFEALVGLCFNEVASFVHGCQNYLRQRREIEGVF-------------SSA
MSTRG.19393.1    TCDVRLIDEGLFEALVALCFNEVASFVHGCQNYLRQRREIEGVPSPGAHGAGGEGATSTS
                 ###########################################                 


                        190       200       210       220       230       240
                 =========+=========+=========+=========+=========+=========+
Ec-15_004080     ATP---PSAAAA--GAATSSSRTKGGG--------GARCESIVSTGGMSAGAGAGGRRKE
MSTRG.19393.1    KTPKPFPSGGAHGFSASRSSPRSKGGGEGGRGGDGGVRSASTISAGETSGVAGAGGRRKE
                                ############        #########################


                        250       260       270       280       290       300
                 =========+=========+=========+=========+=========+=========+
Ec-15_004080     AEGRFTEEKLEVAMDVARTCMAMFAHLTARGHGRRALHAGVLAHINSVPIGAGDDPHMTG
MSTRG.19393.1    AEARFTEEKLEVAMDVARTCMSMFAHLTARGHGRRALHAGVLAYVNSVPIGAGDDPQVTG
                 ############################################################


                        310       320       330       340       350       360
                 =========+=========+=========+=========+=========+=========+
Ec-15_004080     SLSQLVLDLSAVAMEGEENRLSFVGQGPLDAIMRCLRGGRAAQQQCALDALTTVCLLSEM
MSTRG.19393.1    SLSQLVLELSAVAMEGEENRLSFVAQGPLDAIMRCLKGGRAAQQQCALDALKTVCLLTEM
                 ############################################################


                        370       380       390       400       410       420
                 =========+=========+=========+=========+=========+=========+
Ec-15_004080     KPLVFCREVLGEVLMLAAVQEHVQPAAFVLHQFSLLSEWRSPLVRGGCVDLLVAAIAHAT
MSTRG.19393.1    KPLVFCREVLGEVLMLAAVQEHVQSAAFVLHQFSLLPEWRSPLVKGGCLTLLVSAIAHAV
                 ############################################################


                        430       440       450       460       470       480
                 =========+=========+=========+=========+=========+=========+
Ec-15_004080     GPEAPPQEEACPILESAVAAIRQITKLEGAGRVLVDAGGLSQLLRLSRSERENIRRDACQ
MSTRG.19393.1    GPEAPPQEEAFSILDSAVAALRQITKLEGAGRALVEVGGLPQLLRLSRSERNTIRRDACQ
                 ############################################################


                        490       500       510       520       530       540
                 =========+=========+=========+=========+=========+=========+
Ec-15_004080     ALKHIGHRGRDRER---------DHKSESQETVEGSASTD--------------------
MSTRG.19393.1    ALKHIGHKGRDREKKAEGQGLADGHAADATDAAEGGGEVTIEGDTGGVVDGGRDIRSVDL
                 #############           ###########                         


                 
                 ====
Ec-15_004080     ----
MSTRG.19393.1    GDGG
```

```
Parameters used
Minimum Number Of Sequences For A Conserved Position: 2
Minimum Number Of Sequences For A Flanking Position: 2
Maximum Number Of Contiguous Nonconserved Positions: 8
Minimum Length Of A Block: 10
Allowed Gap Positions: None
Use Similarity Matrices: Yes
```

```
Flank positions of the 4 selected block(s)
Flanks: [109  163]  [196  207]  [216  493]  [505  515]  

New number of positions in Ec-15_004080.aa.fasta.trans.aa_ali.fasta-gb:  356  (65% of the original 544 positions)
```
